# Dietary patterns and risk of prostate cancer: a factor analysis study in a sample of Iranian men

**DOI:** 10.15171/hpp.2018.17

**Published:** 2018-04-18

**Authors:** Amir Bagheri, Seyed Mostafa Nachvak, Mansour Rezaei, Mozhgan Moravridzade, Mahmoudreza Moradi, Michael Nelson

**Affiliations:** ^1^Nutritional Science Department, Kermanshah University of Medical Sciences, Kermanshah, Iran; ^2^Student Research Committee, Kermanshah University of Medical Sciences, Kermanshah, Iran; ^3^Department of Biostatistics, Kermanshah University of Medical Sciences, Kermanshah, Iran; ^4^Department of Urology, Kermanshah University of Medical Sciences, Kermanshah, Iran; ^5^Department of Nutrition and Dietetics, King’s College London, London, United Kingdom

**Keywords:** Diet, Dietary patterns, Factor analysis, Prostate cancer, Case–control study

## Abstract

**Background:** Prostate cancer is one of the most common types of cancer with a high mortality rate. The current study was conducted to investigate the relationship between dietary patterns and prostate cancer risk among Iranian men.

**Methods:** This case-control study was conducted in Kermanshah province in western Iran in November 2016. Fifty patients with prostate cancer were selected as cases and 150 healthy men matched for age and body mass index (BMI) were selected as controls. Dietary intake data were collected by a semi-quantitative food frequency questionnaire (FFQ). Food items were grouped according to the similarity of nutrient profiles. The main dietary patterns were identified by factor analysis.

**Results:** After adjustment for potential confounders, a healthy dietary pattern was associated with decreased risk of prostate cancer (highest versus lowest tertile OR:0.24; 95% CI: 0.07-0.81;trend p: 0.025). An unhealthy dietary pattern was related to increased risk of prostate cancer(highest versus lowest tertile OR:3.4; 95% CI: 1.09-10.32; trend p: 0.037).

**Conclusion:** This study shows that an unhealthy dietary pattern was associated with increased risk of prostate cancer. However, a healthy dietary pattern was associated with decreased risk of prostate cancer.

## Introduction

Prostate cancer is one of the most common cancers in males worldwide,^1,2^ and the second leading cause of cancer mortality in men in the United States after lung cancer.^3^ Studies on the epidemiology of prostate cancer in Iran are rare.^4,5^ Prostate cancer is reported as the third most common cancer in men and the sixth most common cancer in Iran.^4^ Incidence of prostate cancer in Iranian men is higher than other countries in Asia.^6^

Clearly identified risk factors for prostate cancer include age, ethnicity, and family history, but the role of other risk factors such as diet, alcohol consumption, exposure to ultraviolet radiation, chronic inflammation, and occupational exposure are less fully investigated.^7^ Diet, for example, can play an important role in incidence of prostate cancer.^8^

Associations between diet and disease risk can be explored in relation to individual nutrients and food consumption and also dietary patterns^9,10^ defined by national or regional eating habits. Prostate cancer incidence has been associated with calcium, green tea, lycopene, pectin, pomegranate, selenium, soy, vitamin D and vitamin E,^1,11,12^ however, this approach to assess nutrients individually has limitations because people eat diets consisting of a variety of foods with complex compounds of nutrients, therefore, interactions and complex inter-relationships between nutrients, cannot be properly recognized.^9,13^ Since the relationship between diet and disease is very complicated, it is suggested that dietary pattern analysis, instead of analyzing a particular nutrient, can be provides more detailed information on the relationship between diet and the risk of disease.^9,14^

A limited number of studies have examined the relationship between dietary patterns and risk of prostate cancer.^13^ While some of them report a positive association between the Western dietary pattern and risk of prostate cancer,^15-18^ other studies show no association.^13,19,20^ On the other hand, some studies show a protective effect of Healthy and Mediterranean dietary patterns on prostate cancer^3,13,15^ but most studies do not find any association between them.^17-20^ And also, information about the role of dietary patterns in developing prostate cancer is limited in Iranian men.

The purpose of the present study is to explore the relationship between dietary patterns and prostate cancer risk among Iranian men.

## Materials and Methods

### Participants and procedures

The study was conducted in Kermanshah province in western Iran in November 2016. Diagnosed prostate cancer patients age 54 to 85 years were identified on admission to a general hospital in Kermanshah. Cancer was confirmed histologically and classified by the Gleason score^18^ in the following percentages: Score 5: 4%, score 6: 42%, score 7: 38%, and score 8: 16%. Patients with a history of any cancer or recent severe weight loss and changes in their diet during last year were excluded from the study, as were patients whose diagnosis of cancer was 6 months ago or more. For the control group, we approached men in Moalem park in Kermanshah where traditionally many elderly and retired people gather. After screening, men with a history of any tumor (benign or malignant) or total prostate specific antigen (PSA) ≥ 3 ng/mL were excluded. Overall, 51 patients with prostate cancer and 160 controls were enrolled in the study. Of these, one case and ten controls withdrew from the study (response rate 94.7%). Cases and controls were matched according to age and body mass index (BMI) (control-to-case ratio = 3:1).

### Measures

Usual dietary intake of the participants during the past year were assessed by using a semi quantitative food frequency questionnaire (FFQ). The FFQ’s validity and reliability have been confirmed previously.^21^ The FFQ includes 147 items with a standard serving size commonly consumed by Iranians. Participants were asked to report their consumption frequency of each food item on a daily, weekly, monthly and yearly basis. The reported frequency for each food item was then converted to a daily intake. Portion sizes of consumed foods in household measures were converted to grams.^22^ Energy and nutrient content of foods were computed by the Nutritionist 4 software based on United States Department of Agriculture (USDA) food composition table modified for Iranian foods.^15,23^

General questionnaires were used to collect sociodemographic characteristics of participants and information about lifestyle that included age (years), smoking history (yes or no), alcohol drinking history (yes or no), medical history (cardiovascular, diabetes, kidney disease and no disease), education (less than a high school diploma, high school diploma or more), socioeconomic status (based on income) and family history of cancer (yes or no). Physical activity levels in this study were determined by interview using the International Physical Activity Questionnaire (IPAQ).^24^ Frequency and duration of physical activity were then expressed as metabolic equivalent of tasks (METs). Weight and height measures were assessed while the participants had the light clothes and were bare feet via a digital scale to the nearest 100-grams accuracy and a stadiometer to the nearest 0.1 cm. PSA concentrations in serum samples were measured by radioimmunoassay technique (Pars, Kermanshah, Iran). The laboratory is under the supervision of Kermanshah University of Medical Sciences. 

### Statistical analysis

In order to reduce the complexity of the data, the 147 FFQ food items were categorized into 24 food groups (Table 1). Food items were grouped according to the similarities in their nutrient profiles. The major dietary patterns were identified by principal component analysis. Varimax rotation was used to create a simple matrix for better interpretation and identify uncorrelated dietary patterns (factors). Scree plots were used to determine the number of dietary patterns. Two major dietary patterns were selected with eigenvalues greater than 1.7. According to the intake of food groups weighted by the factor loadings, each participant received a factor score for each dietary pattern. Factor loadings were equal to the correlation coefficients between food groups and dietary patterns.^25^ We categorized participants by tertiles of dietary pattern scores. We used the chi-square test to check the differences of distribution of qualitative variables (e.g. family history of cancer, medical history, smoking, alcohol dinking, physical activity and level of education). To evaluate continuous variables, the Kolmogorov–Smirnov test was initially used to assess the normality assumption, if the quantitative data were normal, the independent sample *t* test was used and if the quantitative data were not normal, the Man-Whitney U-test was used to evaluate the differences between 2 groups. Unconditional logistic regression was used to estimate odds ratio (OR) with 95% CI. Physical activity (inactive, moderately active and active), total energy intake, alcohol consumption, smoking and educational level were considered as potential confounders and were included in the models. Data were analyzed with Statistical Package Software for Social Science, version 16 (SPSS Inc., Chicago, IL, USA), and a *P* value of 0.05 was considered significant.

## Results

Using factor analysis, 2 major dietary patterns were identified: an “unhealthy” dietary pattern characterized by higher consumption of hydrogenated fats, refined grains, butters, red meats, process meats, organ meats, sweets, desserts, soft drinks, salt, high-fat dairy, snacks, tea and coffee; and a “healthy” dietary pattern characterized by higher consumption of legumes, fish, low-fat dairy, fruits and fruit juices, vegetables, poultry, nuts and eggs. The factor-loading matrixes for these dietary patterns are shown in Table 2. Factor loading represents the correlation coefficient between a food group with each pattern. Larger values indicate stronger correlations, and positive or negative sign indicates direct or inverse relationships between that group and the pattern. These 2 major dietary patterns explained 21.4% of the total variance in dietary intakes.

Characteristics of participants in relation to prostate cancer are presented in Table 3. Statistically significant differences between cases and controls were observed for calorie intake (*P *= 0.02), physical activity (*P *< 0.001), drinking alcohol (*P *= 0.002) and smoking (*P *= 0.019).

OR and CIs for risk of prostate cancer by tertiles of food pattern scores are shown in Table 4. After adjusting for the potential confounding effects of alcohol consumption (yes or no), smoking (yes, no), total energy intake, physical activity level and educational level, the healthy dietary pattern was associated with a decreased risk of prostate cancer (highest versus lowest tertile OR: 0.24; 95% CI: 0.07-0.81; trend *P *= 0.025). The unhealthy dietary pattern was associated with an increased risk of prostate cancer (highest versus lowest tertile OR: 3.4; 95% CI: 1.09-10.32; trend *P *= 0.037).

## Discussion

Cancer is a major health problem for Iran and rates are increasing.^4^ Unfortunately, unlike developed countries, Iranian treatment and management of cancer is challenging. The majority of Iranian patients with cancer are faced with an unpleasant and tragic end; cancer takes both their lives and their money. The best strategy, therefore, is to focus on the prevention of disease. Food plays an important role in the prevention (and progression) of a variety of diseases, including cancer.

The relationship between intakes of specific nutrients and many cancers has been widely studied. According to fact that the diets include a variety of foods with complex combinations of nutrients interactions and complex inter-relationships between nutrients, cannot be properly recognized.^9,14^ Therefore, it is suggested that dietary pattern analysis, instead of analyzing a particular nutrient, can provide more detailed information on the relationship between diet and the risk of disease.^9,14^Attention to food groups rather than singular nutrients is more likely to reflect the usual dietary intake, and represent features of the total diet, including all nutrient interactions.^14^ And also, the determination of dietary patterns and their relationship to disease can give us the clearer and applicable approach to deal with the disease.

Factor analysis is one of the most reliable methods to identify dietary patterns.^26^ In this study, by using factor analysis, 2 major dietary patterns were identified. The first pattern was termed an “unhealthy”, characterized by diets high in red and organ meat, saturated fats, high-fat dairy products, refined grains, sweets and desserts, salt, and soft drinks, and low in whole grains and unsaturated fat. The second pattern was termed “healthy”: diets were typically higher in legumes, fish, low-fat dairy products, fruits, vegetables and eggs. The unhealthy pattern can be described as energy dense, and the healthy pattern as nutrient dense. There was an inverse relation between the healthy pattern and risk of prostate cancer; the unhealthy pattern, in contrast, was associated with increased risk of prostate cancer.

The present findings are consistent with a number of other studies. In a study in Tehran of 50 men and 100 controls, 2 dietary patterns were identified: “Western diet” (including higher levels of consumption of sweets, desserts, total meat, tea and coffee, French fries, salt, soft drinks, red meat or processed meat) and “healthy diet” (including higher levels of consumption of legumes, fish, dairy products, fruits and fruit juices, vegetables, boiled potatoes, cereals and eggs). The study showed that the western dietary pattern was associated with a significantly increased risk of prostate cancer (OR = 4.0, 95% CI = 1.5-11.0); the healthy dietary pattern was associated with a decreased risk of prostate cancer (OR = 0.4, 95% CI = 0.2-1.0).^15^ In a case-control study of diet and prostate cancer in Australia, three dietary patterns were identified: vegetarian, western and health-conscious. The western diet (with higher consumption levels of red and processed meat, fried fish, chips, hamburgers, white bread and whole milk was associated with higher risk of prostate cancer (1.82, 95% CI**= 1.15-2.87, trend *P *= 0.02).^16^

Not all studies have shown this association. Muller and colleagues, for example, identified four dietary patterns among people with prostate cancer. These were (*a*) Mediterranean (high intake of meats, vegetables, and fruits, and low intake of cakes and sweet biscuits); (*b*) Vegetable (high intake of vegetables); (*c*) Meat & Potatoes (high intake of meats and potato); and (*d*) Fruit & Salad (high intake of salad greens and fruit). They did not, however, find any association between these patterns and risk of prostate cancer.^20^ Extensive overlap of dietary patterns with each other may be one of the reasons that no associations between dietary patterns and prostate cancer were observed. In another study, 2 dietary patterns, “prudent” (which includes high intake of fruits, vegetables, whole grains, fish and poultry) and “western” (which contained a lot of red and processed meat, refined grains and high fat dairy products) were identified.^27^ The “western” dietary pattern was associated with an increased risk of prostate cancer, but this did not reach statistical significance, due possibly limitations in their study.

Taking into account race, culture and place, 2 dietary patterns associated with risk of prostate cancer have been identified: a healthy pattern that includes higher consumption levels of fruits and vegetables, legumes, whole grains, low-fat dairy, fish and poultry; and an unhealthy pattern that includes higher consumption levels of red and processed meat, refined grains, sweets, high-fat dairy and fried foods. Pathogenicity of the unhealthy diet patterns can be attributed to factors such as heterocyclic amines,^8,28^ oxidizing agents,^8,29^ anabolic agents,^30-32^ high-calorie,^33^ high-fat^19,34^ and lack of the antioxidants.^35^

The main limitation of this study was the use of food frequency questionnaires for dietary assessment. Although we used a validated questionnaire, it can still over- or under-estimate consumption of specific foods. In factor analysis, the researcher can make subjective decisions that can impact on results and interpretation.^9,19^ In this study, the 2 patterns were based on eigenvalues, scree plots, and interpretability to find significant patterns. Low sample size was another limitation of this study. And therefore, it was not possible to adjust the results for confounder effects of vitamins and mineral supplementation because information about this exposure was not collected at baseline. Because of the limitations of case-control studies, as well as limited studies conducted on the dietary pattern and the risk of prostate cancer in Iran, it is suggested that cohort studies be conducted in this regard.

An important issue in relation to cancer is assessment of exposure to toxins and various chemical compounds, or chemical pollution among the subjects. In Iran, use of pesticides and fertilizers, particularly nitrate fertilizers, are common.^36^ Some pesticides are non-standard and potentially dangerous, with poor control over their use. These toxins, pesticides and fertilizers can easily enter the food chain.^37^ Information on their levels of ingestion in Iran is sparse.

In conclusion, we found 2 major healthy and unhealthy dietary pattern which was associated with lower and higher risk of prostate cancer, respectively.

## Ethical approval

Ethical approval was given by the Ethics Committee of the Deputy of Research and Technology of Kermanshah University of Medical Sciences (ethic number: KUMS.REC.1395.6).

## Competing interests

The authors declare that they have no competing interests.

## Authors’ contributions

AM contributed to the design of the study, data collection, analysis and interpretation of data and drafting of the manuscript. SMN contributed to the design of the study, analysis and interpretation of data, the management, appraised and revised of the manuscript. MR helped us in statistical analysis. MM helped us in data collection. MRM was involved in the design of the study. MN was involved in the conception of the study and assisted revision. All authors approved the final version of the manuscript for submission.

## Acknowledgments

We thank the participants involved in our study. The author gratefully acknowledges the Research Council of Kermanshah University of Medical Sciences (grant number: 95021) for financial support. This work was performed in partial fulfillment of requirements for (master of sciences in nutrition, D) of (Amir Bagheri), in the faculty of Nutritional Science & Food Technology, Kermanshah University of Medical Sciences, Kermanshah, Iran.

**Table 1:** Food groupings used in the dietary pattern analyses

**Food groups**	**Food items**
Refined grains	White breads (lavash, baguettes), noodles, pasta, rice
Whole grains	Dark breads (Iranian),‏ corn, barley
Legumes	Beans, peas, broad beans, lentils, split pea, mung bean, soy
Red meats	Beef, lamb, minced meat
Processed meats	Hamburger, sausage, delicatessen meat, pizza
Organ meats	Heart, kidney, liver, tongue, brain, offal
Poultry	Chicken
Fish	All fish types
Eggs	Eggs
Low-fat dairy products	Low-fat milk, low-fat yogurt, yogurt drink (*doogh*)
High-fat dairy products	High-fat milk, whole milk, chocolate milk, cream, high-fat yogurt, cream cheese, other cheeses, ice cream
Vegetables	Cauliflower, carrot, tomato and its products, spinach, lettuce, cucumber, eggplant, onion, greens, green bean, green pea, squash, mushroom, pepper, garlic, turnip, others
Pickles	Pickles
Fruits and fruit juices	Melon, watermelon, honeydew melon, plums, prunes, apples, cherries, sour cherries, peaches, nectarine, pear, fig, date, grapes, kiwi, pomegranate, strawberry, banana, persimmon, berry, pineapple, oranges, dried fruits, all juices, others
Soft drinks	Soft drinks
Hydrogenated fats	Hydrogenated fats, animal fats
Butters	Butter, margarine, mayonnaise
Vegetable oils	Vegetable oils, olive oils
Nuts	Almond, peanut, walnut, pistachio, hazelnut, seeds
Sweets and desserts	Cookies, cakes, biscuit, muffins, pies, chocolates, honey, jam, sugar cubes, sugar, candies, others
Tea and coffee	Tea and coffee
Salt	Salt
Snacks	Corn puffs, potato chips, French fries
Condiments	Condiments

**Table 2:** Factor-loading matrix for 2 dietary patterns

**Food groups**	**Dietary patterns**	
	**Unhealthy**	**Healthy**
Hydrogenated fats	0.655	-0.203
Salts	0.560	
Butters	0.524	
Refined grains	0.510	
Red meats	0.479	
Sweets and desserts	0.413	
Vegetable oils	-0.394	
Soft drinks	0.388	
Snack	0.380	0.230
Organ meats	0.344	
High-fat dairy	0.341	0.277
Whole grains	-0.242	
Tea and coffee	0.231	
Processed meats		
Vegetables		0.688
Fruit and fruit juices		0.657
Fish		0.547
Legumes		0.476
Poultry		0.440
Nuts		0.391
Eggs		0.326
Condiments		0.325
Low-fat dairy		0.294
Pickles		0.272
Variance %	11.1	10.3

Values < 0.20 have been removed for clarity.

**Table 3:** General characteristic of the participants in the study

	**Cases** **No. (%)** **n = 50**	**Controls** **No. (%)** **n = 150**	***P*** ** value**
Age	69.7 (7.3) ^*^	67.9 (7.7)^*^	0.147 ^a^
BMI	24.64 (3.4) ^*^	24.65 (3.3) ^*^	0.97^a^
WHR	0.95 (0.05) ^*^	0.93 (0.09) ^*^	0.167 ^a^
Total calorie intake	2543 (383) ^*^	2332 (602) ^*^	0.02 ^a^
Socioeconomic status			0.071^b^
Low income	25 (50)	48 (32)	
Middle income	21 (42)	89 (59.3)	
High income	4 (8)	13 (8.7)	
Family history of cancer			0.251^b^
Yes	15 (30)	33 (22)	
No	35 (70)	117 (78)	
Medical history			0.563 ^b^
CVD	18 (36)	51 (34)	
DM	5 (10)	13 (8.7)	
Kidney disease	5 (10)	7 (4.7)	
None	22 (44)	79 (52.6)	
Smoking			0.019 ^b^
Yes	23 (46)	42 (28)	
No	27 (54)	108 (72)	
Alcohol drinking			0.002 ^b^
Yes	16 (32)	19 (12.7)	
No	34 (68)	131 (87.3)	
Physical activity			< 0.001 ^b^
Low	35 (70)	63 (42)	
Middle	15 (30)	61(40.7)	
High	0	26 (17.3)	
Education			0.054 ^b^
Low	34 (68.0)	73 (48.7)	
Middle	13 (26.0)	58 (38.7)	
High	3 (6.0)	19 (12.7)	

Abbreviations: BMI, body mass index; WHR, waist to hip ratio; CVD, cardiovascular disease; DM, diabetes mellitus. * Mean ± SD; all other data reported as number (%). ^a^Data analyzed by student *t* test. ^b^Data analyzed by chi-square.

**Table 4:** Adjusted and unadjusted odds ratios and 95% CI for the risk of prostate cancer associated with dietary patterns identified in the study

	**Unhealthy pattern**				**Healthy pattern**			
	**T1** ^a^	**T2**	**T3**	***P***_trend_	**T1**	**T2**	**T3**	***P***_trend_
Case	6	16	28	0.037	21	20	9	0.025
Control	60	51	39	0.037	45	47	58	0.025
OR (95% CI)^b^	1	2.8 (1.08-7.78)	6.9 (2.58-18.47)	<0.001	1	1.01 (0.46-2.21)	0.36 (0.14-0.90)	0.035
OR (95% CI)^c^	1	2.2 (0.75-6.97)	3.4 (1.09-10.32)	0.037	1	0.71 (0.29-1.74)	0.24 (0.07-0.81)	0.025

^a^ Tertile 1,2,3. ^b^ Crude OR. ^c^ Adjusted for alcohol consumption, smoking, total energy intake, physical activity and educational level.
